# MMR Deficiency Defines Distinct Molecular Subtype of Breast Cancer with Histone Proteomic Networks

**DOI:** 10.3390/ijms24065327

**Published:** 2023-03-10

**Authors:** Sean Hacking, Charissa Chou, Yigit Baykara, Yihong Wang, Alper Uzun, Ece D. Gamsiz Uzun

**Affiliations:** 1 Department of Pathology and Laboratory Medicine, Rhode Island Hospital, Providence, RI 02903, USA; 2 Department of Pathology and Laboratory Medicine, Warren Alpert Medical School of Brown University, Providence, RI 02903, USA; 3 Department of Biology, Brown University, Providence, RI 02912, USA; 4 Legorreta Cancer Center, Brown University, Providence, RI 02912, USA; 5 Department of Pediatrics, Warren Alpert Medical School of Brown University, Providence, RI 02903, USA; 6 Center for Computational Molecular Biology, Brown University, Providence, RI 02912, USA

**Keywords:** MMR, breast cancer, comprehensive genomic profiling, Proteinarium, histone proteins

## Abstract

Mismatch repair (MMR) alterations are important prognostic and predictive biomarkers in a variety of cancer subtypes, including colorectal and endometrial. However, in breast cancer (BC), the distinction and clinical significance of MMR are largely unknown. This may be due in part to the fact that genetic alterations in MMR genes are rare and only seen to occur in around 3% of BCs. In the present study, we analyzed TCGA data using a multi-sample protein–protein interaction (PPI) analysis tool, Proteinarium, and showed a distinct separation between specific MMR-deficient and -intact networks in a cohort of 994 BC patients. In the PPI networks specific to MMR deficiency, highly connected clusters of histone genes were identified. We also found the distribution of MMR-deficient BC to be more prevalent in HER2-enriched and triple-negative (TN) BC subtypes compared to luminal BCs. We recommend defining MMR-deficient BC by next-generation sequencing (NGS) when any somatic mutation is detected in one of the seven MMR genes.

## 1. Introduction

Worldwide, a woman is diagnosed with breast cancer (BC) every 9 s [1]. Mismatch repair (MMR) is important in daily clinical practice for cancer management in a variety of cancers, including colon and endometrium, as screenings are used to identify the potential of Lynch syndrome [2]. Today, the role of MMR in BC is not well understood. An early report [3] on 16 BC patients demonstrated no incidence of MMR in five microsatellite loci (D2S123, D3S1611, D17S807, D17S796, and Xq11-12), initially suggesting that MMR is uncommon in human early-onset BC and does not appear to be related to prognosis. In BC, most clinical emphasis is placed on a patient’s individual hormone receptor (HR) status (estrogen receptor (ER), progesterone receptor (PR), and human epidermal growth factor receptor 2 (HER2)) as well as the tumor proliferative index measured by Ki-67. Roughly, this allows tumors to be subdivided into the following classical molecular subtypes: Luminal A (ER+/-, PR+/-, HER2-), Luminal B (ER+/-, PR+/-, HER2+, or Ki67 > 14–20%), HER-2-positive (ER-, PR-, HER2+), and triple-negative (TN)/basal (ER-, PR-, HER-2-) [4]. A normal-like subtype has also been described; although its significance is still unclear, it is characterized by normal breast tissue profiling [5].

MMR encompasses the post-replication preservation of DNA homeostasis and genomic stability [6]. The MMR system functions not only to correct spontaneous base–base mispairing, but also small insertions–deletions (indels) that occur following DNA replication; subsequent failure results in an increased mutation rate [6]. The MMR system contains a group of genes which encode proteins interacting as heterodimers. In humans, there are eight genes which make up the MMR machinery and encode for its protein components. These include the homologs of *E. coli* MutS genes MSH2, MSH3, MSH5, and MSH6, and the MutL genes MLH1, PMS1 (MLH2), MLH3, and PMS2 (MLH4) [6,7,8]. Mutations in MMR can lead to a loss of translation and subsequently genomic instability. However, MSH5 has not been found to be associated with MMR in humans [6]. MSH2 is located on chromosome 2p21 and is considered the principal MSH protein which creates distinct heterodimers with MSH6 and MSH3, referred to as MutSα and MutSβ, respectively [9]. MLH1 encodes a protein that dimerizes with PMS2 to form MutLα and MLH3 which forms MutLβ [9].

Regarding research progress in MMR and breast cancer, data on both prevalence and prognostic significance are limited. Fusco et al. showed that luminal B-like MMR-deficient breast carcinomas have a shorter overall survival than those with proficient MMR, while patients with ER-negative BC treated with chemotherapy lived longer with MMR deficiency [10]. Other studies have shown MMR to predict poor short-term survival in breast cancer patients treated with chemotherapy and have found associations between MMR, loss of heterozygosity, and the occurrence of secondary breast tumors [11,12,13].

Most recently, genome-wide association studies (GWASs) have grown in popularity as they offers an approach to investigate complex diseases such as BC [14]. However, GWASs have fallen short in identifying ‘missing heritability’, as our fundamental understanding of the complex architecture of the genome due to additive genetic effects is not fully represented by GWASs [15]. Analyzing the complex architecture could demonstrate subgroups of patients who share variants of genes in specific networks with a shared phenotype. To combat this problem, we developed Proteinarium, a multi-sample protein–protein interaction (PPI) tool with the ability to identify clusters of cancer patients with shared gene networks [15]. Proteinarium functions by converting user-defined seed genes into corresponding protein symbols, which are then mapped onto the STRING database’s interactome [16]. A PPI network is built for each sample using Dijkstra’s algorithm [17]. Pairwise similarity scores are calculated to compare the networks and cluster the samples where they are presented as a dendrogram. A layered graph of PPI networks for the samples in any cluster can be visualized and analyzed.

The principle aims of this study are to determine whether MMR-deficient BC is associated with a distinct molecular subtype, identify unique PPI networks, and finally compare MMR across molecular BC subtypes. This could allow for a better understanding, demonstrate significance, and facilitate a better, more standardized definition of MMR deficiency in BC.

## 2. Results

### 2.1. Mutational Portraits of MMR Genes Signatures in Human Breast Cancer

A total of 994 patients were included based on the availability of sequencing data, and 89 were found to have mutations including VUSs, structural variants, or copy number alterations (CNAs) in any of the seven MMR genes: MSH2, MSH3, MSH6, MLH1, PMS1, MLH3, and PMS2. Excluding VUSs, our cohort consisted of 29 patients with MMR deficiency (2.9%). MSH2 had a somatic frequency of 1.0% with four driver mutations: one truncating, one splice, and two SV/fusion. MSH3 had a somatic mutation frequency of 0.7% with three driver mutations, all truncating, and five VUSs, all of which were missense. Seven CNAs were seen for MSH3 with homozygous deletion. MSH6 had a somatic mutation frequency of 0.9%: two truncating, one SV/fusion, and eight missense variants which were VUSs. MLH1 had a somatic mutation frequency of 0.9% with five driver mutations—one missense, two truncating, one splice, and one SV/fusion—and five VUSs, all of which were missense. Four CNAs were seen for MLH1 with homozygous deletion. One patient had both an MLH1-GOLGA4 fusion and a CNA. PMS1 had a somatic mutation frequency of 1.2% with three driver mutations: two truncating and one SV/fusion; nine VUSs were seen, all missense. One CNA was seen for PMS1 with homozygous deletion. MLH3 had a somatic mutation frequency of 1.1% and contained 12 VUSs, 11 of which were missense and 1 inframe. PMS2 had a somatic mutation frequency of 0.8% and contained two driver mutations, all truncating, and six VUSs, all missense. One CNA was seen for PMS2, homozygous deletion (Supplementary Table S1).

The MMR-deficient cluster identified in Proteinarium included 17 patients out of 29 MMR-deficient patients. MSH2 had a somatic frequency of 11.8% with two driver mutations: one SV/fusion and one splice. MSH3 had a somatic mutation frequency of 5.9% with one driver truncating mutation. MSH6 had a somatic mutation frequency of 11.8% with two driver mutations: one truncating and one SV/fusion. MLH1 had a somatic mutation frequency of 11.8% with two driver mutations: one missense and one splice. PMS1 had a somatic mutation frequency of 5.9% with one driver mutation, truncating. MLH3 had a somatic mutation frequency of 0%. PMS2 had a somatic mutation frequency of 5.9% and contained one driver mutation, truncating. Regarding molecular subtype, 14 patients were basal (TN), 2 were HER2-enriched, and 1 was Luminal B. The MMR-deficient cohort had good overall survival metrics with a 77.92% disease-free survival (mean time to event of 2.9 years), 73.05% progression-free survival (mean time to event of 2.5 years), 85.23% overall survival (mean time to event of 2.5 years), and 85.23% disease-specific survival (mean time to event of 2.5 years).

### 2.2. MMR-Deficient and MMR-Intact Patients Have Distinct PPI Networks

**Figure 1:**
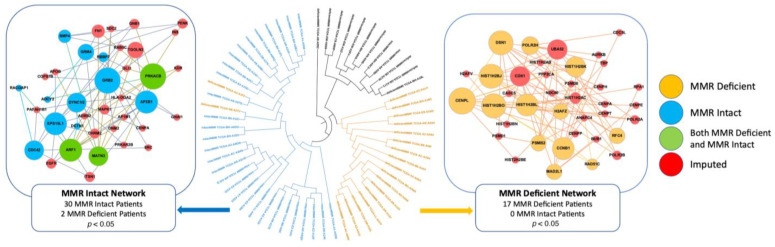
Dendrogram and consensus networks of specific MMR-intact (left) and MMR-deficient clusters (right) Larger nodes indicate higher levels of connectivity. Patients with blue branches contribute to the MMR-intact consensus network (left). Patients with orange branches are part of a significant cluster that create the MMR-deficient consensus network (right). Orange nodes denote genes unique to MMR-deficient patients, blue nodes denote genes unique to MMR-intact patients, green nodes denote genes present in both MMR-deficient and MMR-intact patient networks for that cluster, and red nodes denote imputed genes. ITOL was used to generate the circular dendrogram [18].

The Proteinarium dendrogram revealed significant clusters (*p* < 0.05) dominated by either MMR-deficient or MMR-intact patients. Two clusters were chosen for further investigation based on the degree of domination by either MMR-deficient or MMR-intact patients and the number of patients in the cluster. Excluding VUSs, the MMR-deficient cluster contained 17 MMR-deficient patients and 0 MMR-intact patients, while the MMR-intact cluster contained 30 MMR-intact patients and 2 MMR-deficient patients (Figure 1) [18]. The consensus networks for significant clusters dominated by MMR-deficient and MMR-intact patients include genes unique to each MMR status group.

The hub or most connected proteins exclusive to the MMR-intact patient networks include GRB2, AP2B1, DYNC1I2, EPS15L1, and CDC42, with GRB2 being the most highly connected protein in the network. GRB2 is an adaptor protein that critically links growth factor receptors on the cell surface to the Ras signaling pathway, which promotes cell growth, survival, and differentiation [19]. A subnetwork of histone proteins was found in the MMR-deficient consensus network. These histone proteins, which are unique to the MMR-deficient patients, are members of the histone H2B family. CENPL and CCNB1 are two other hub proteins unique to the MMR-deficient network. CENPL is a subunit of the centromeric complex required for proper kinetochore function and the progression of mitosis [20]. CCNB1 encodes Cyclin B1 and is necessary for the regulation of the G2/M phase transition of the cell cycle [21]. Both CENPL [22] and CCNB1 [23] have been found to be upregulated in breast cancer tissues. CCNB1 overexpression has been found to be associated with worse outcomes in triple-negative breast cancer [24,25].

The s_AB_ distance between the MMR-deficient and MMR-intact consensus networks was calculated as 0.324 for the comparison. The positive values of the separation score s_AB_ indicate that the networks do not overlap in the interactome and can be considered as distinct molecular entities.

### 2.3. Molecular and Clinicopathologic Profile

**Figure 2:**
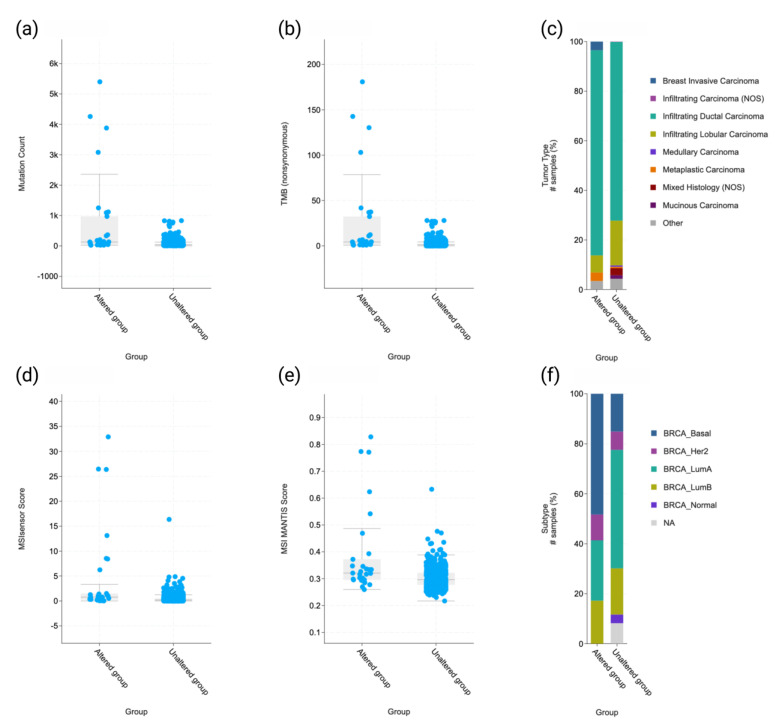
Molecular and clinicopathologic comparisons between MMR-mutated and -intact groups excluding VUSs. (**a**) Higher mutation count (*p* = 1.3 × 10^−7^), (**b**) higher tumor mutational burden (TMB) (*p* = 3.3 × 10^−7^), (**c**) invasive ductal carcinoma tumor histology type (*p* = 2.3 × 10^−6^), (**d**) higher MSIsensor score (*p* = 8.2 × 10^−6^), (**e**) MANTIS score (*p* = 1.3 × 10^−4^), and (**f**) basal/TN molecular subtype status (*p* = 2 × 10^−4^).

Molecular, clinical, and pathologic findings were compared with MMR status on the cBioPortal. An overview of MMR mutations and the patient cohort is available in Supplementary Table S2. In the 29 patients excluding VUSs, MMR was found to be associated with a higher mutation count (*p* = 1.29 × 10^−7^), higher tumor mutational burden (TMB) (*p* = 3.27 × 10^−7^), invasive ductal carcinoma tumor histology type (*p* = 2.29 × 10^−6^), higher MSIsensor score (*p* = 8.18 × 10^−6^), MANTIS score (*p* = 1.28 × 10^−4^), and basal/TN molecular subtype status (*p* = 2.1 × 10^−4^) (Figure 2, Supplementary Table S3).

In the MMR-deficient cohort, Luminal B had worse progression-free survival (*p* = 7.0 × 10^−3^), overall survival (*p* < 1.0 × 10^−10^), and disease-specific survival (*p* < 1.0 × 10^−10^). HER2 subtype cancers were found to have worse progression-free survival (*p* = 5.0 × 10^−3^), overall survival (*p* = 5.1 × 10^−4^), and disease-specific survival (*p* = 7.9 × 10^−5^). In the larger cohort of MMR-deficient patients including VUSs, HER2-enriched tumors were found to have worse disease-free survival (*p* = 0.049), progression-free survival (*p* = 7.2 × 10^−3^), and disease-specific survival (*p* = 0.029). MMR deficiency in this larger cohort also found the TN/basal subtype cancer to have better overall survival (*p* = 0.034) (Supplementary Figure S1).

## 3. Discussion

Alterations in MMR mechanisms have been found to be relevant to human carcinogenesis since 1993 when the replication errors (RER) phenotype was discovered in bacteria [26]. MMR is seen to be particularly relevant in colorectal [27] and endometrial [28] carcinomas. Most recently, the use of immune checkpoint inhibitors has been approved and is seen to be appropriate in all MMR-deficient cancers [29]. However, in the clinical management of breast cancer, immune checkpoint inhibitors are used almost exclusively in patients with a TN/basal biomarker status [30].

The clinical findings in the present study support that MMR mechanisms are distinct depending on a tumor’s hormone receptor status and corresponding breast cancer molecular subtype. Fusco et al. demonstrated that breast cancer patients who were Luminal B-like and MMR-deficient showed shorter overall survival than those who were MMR-intact [10]. On the other hand, they found patients with ER-negative breast cancers treated with chemotherapy to live longer with MMR deficiency. The findings in the present study also support the association of TN/basal molecular status and MMR deficiency with improved survival including VUSs and worse survival in Luminal B- and HER2-enriched patients. Some possible explanations for improved survival with MMR deficiency in TNBC include: (1) MMR deficiency was most common in these tumors, (2) immune check point inhibitors are commonly used in this cancer subtype, and (3) MMR-deficient tumors have been shown to respond to checkpoint point inhibition across many cancer subtypes, although breast was not studied [31]. Cheng et al. also found MMR deficiency to be significantly associated with worse overall and disease-specific survival in ER-positive breast cancer patients who were all treated with tamoxifen as an isolated adjuvant systemic therapy [32]. However, there were 31 MMR-deficient patients identified by IHC in the study [32]. In the present study, there were 29 MMR-deficient patients, and shorter survival was demonstrated in patients with HER2 molecular breast cancer status who were MMR-deficient.

We showed histone-related proteins as hub proteins in MMR-deficient patients, particularly in the MMR-deficient consensus network. DNA MMR is found to be less efficient in targeting and replacing mispairs packaged in chromatin, as MMR must either compete for access to naked DNA before histone deposition or actively move nucleosomes [33]. Previous studies have demonstrated that neither purified MMR proteins nor nuclear extracts of human cells can repair DNA mismatches in the context of chromatin in vitro [34,35]. Li et al. have further postulated that this may be due to the chromatin histone structure itself inhibiting communication between the mismatch and nick site or that MutS may not efficiently recognize DNA mispairs when bound by a histone octamer [36]. Recently, it has been suggested that the MutSα interaction with the chromatin H3K36me3 active histone mark may also play a role in promoting MMR activity at sites of transcription [37]. Regarding the interactive complexity of the chromatin landscape, HUB2 gene signatures have been found to antagonize H3K27me3 marks in plant growth and development [38]. HUB2 has been shown to increase histone H2B monoubiquitination in eukaryotes [39]. The MMR-deficient cluster of patients appears to be a favorable, predominantly ductal BC subtype associated with TN molecular status. The association with radiation status was likely secondary to the fact that these tumors were predominantly TN (54%), and TN status is associated with higher radiation therapy (RT) utilization at 69.2% compared to 55.7% in ER+/HER2+, 57.1% in ER+/HER2-, and 65.6% in ER-/HER2+ patients [40].

Further studies examining MMR in relation to histone proteins and epigenetic modifications generally in TNBC could be beneficial. Recently histone lysine methyltransferases (KMTs) have emerged as attractive drug targets in BC, although therapies targeting histone modifications (HMs) are still in the initial phases [41]. KMT nuclear receptor binding SET domain protein 2 (NSD2) has been shown to be overexpressed in TNBC tumors and to control the expression of EGFR and ADAM9, a member of the ADAM (a disintegrin and metalloproteinase) family which releases growth factors including HB-EGF [42]. NSD2 may be identified as a major epigenetic regulator in TNBC.

The possible concordance between MMR by immunohistochemistry (IHC) and NGS may explain some discrepancies between the two previous studies and this study [10,32]. Importantly, Fusco et al. showed the rate of discrepancy between IHC and molecular MSI analysis to be very high (91%) [10]. In the study, the data were obtained and no definitive IHC was performed to examine protein expression. Validating IHC and its relationship to NGS results will be important for guiding diagnostic MMR workflows in BC. Current trends are moving away from traditional IHC and towards more comprehensive molecular profiling. The evaluation of any mutation detected by NGS in any of the seven MMR deficiency genes found in our study may be appropriate.

In November 2020, the U.S. Food and Drug Administration (FDA) approved Keytruda (pembrolizumab) in combination with chemotherapy for unresectable locally advanced or metastatic TN, PD-L1-positive breast cancers based on the results of the KEYNOTE-355 clinical trial [43]. More recently, MMR has been postulated as a molecular target in TNBC precision oncology, harboring an increased sensitivity to immunotherapy [44]. MMR testing could be used in clinical practice to help guide treatment based on immunotherapy, particularly in HER2-enriched and TN tumors, as we found these to involve MMR more proportionally. Additionally, mutational signatures associated with MMR have been found to be enriched in breast cancer brain metastases compared to primary breast tumors [45], suggesting that there may be a role of MMR and PD-L1 testing in metastatic breast cancer specimens.

It is important to mention the numerous pitfalls in the present study, including that we were unable to determine immune cell density and phenotypes in the tumoral microenvironment. MMR-deficient tumors often exhibit a high mutational burden and express neoantigens generated by frameshift mutations in coding microsatellites, which stimulate lymphocytic infiltration as well as the up-regulation of inflammatory cytokines [46].

Finally, it would have been beneficial to validate the findings of our study in a second independent breast cancer cohort. The primary aim of our study is secondary data analysis, but as a future reference, it would also be beneficial to perform an experimental validation of these findings.

## 4. Materials and Methods

### 4.1. Study Design

This study included cases from the Breast Invasive Carcinoma dataset (TCGA, PanCancer Atlas) [47,48,49,50,51,52,53,54,55,56]. The RNA-seq data were obtained from the cBioPortal (https://bit.ly/3g4lTM4) (accessed on February 1, 2022). Tumor data were obtained from patients that had not received prior treatment for their disease (ablation, chemotherapy, or radiation therapy). The initial cohort included 1084 patients. Cases without any genomic data in the following genes were excluded: MSH2, MSH3, MSH6, MLH1, PMS1, MLH3, and PMS2. The final cohort included 994 patients.

### 4.2. Proteinarium

Proteinarium, a novel multi-sample tool used to identify clusters of patients with shared PPI networks, was used to compare MMR-deficient and MMR-intact patients. MMR deficiency was defined as having a mutation (excluding VUS) in one or more of the seven MMR machinery genes (MSH2, MSH3, MSH6, MLH2, PMS1, MLH3, or PMS2). Based on this criterion, 29 patients were defined as MMR-deficient. Proteinarium uses the experimentally validated PPI information from the STRING database to build network graphs based on the seed genes for each patient. The similarity between each pair of network graphs is calculated using the Jaccard distance, which is recorded in a similarity matrix used as an input for clustering graphs. Unweighted pair group method with arithmetic mean (UPGMA) is used to cluster patients, with their network similarities as an output. The significance of a cluster is calculated using Fisher exact tests, based on the abundance of cases and controls. The minimum path length was set at 2 and thus included only the pathways of seed proteins connected directly to each other and/or via a single intermediary protein (“imputed genes”).

To choose the number of seed genes for each patient, we calculated the cophenetic correlation coefficients to compare the dendrograms for the Proteinarium runs. The runs included 29 MMR-deficient and 965 MMR-intact patients. The top 60, 100, 150, 200, 250, and 300 most upregulated genes were ranked based on the z-scores for the genes in each patient and used in the Proteinarium runs. Cophenetic correlation coefficients require that the patients in all compared dendrograms are exactly the same. Cophenetic correlation coefficients measure the degree of similarity between two dendrograms, with values closer to 1 indicating more similarity in the dendrogram structure. The cophenetic correlation coefficients of the top 60, 100, 150, 200, 250, and 300 genes were compared (Supplementary Figure S2). 150 was chosen as the number of seed genes.

We performed random iterations of the Proteinarium runs using the top 150 seed genes for each patient. Random iterations were necessary because the number of MMR-intact patients (965 MMR-intact patients) was much greater than the number of MMR-deficient patients (29 MMR-deficient patients). The workflow for the random iteration process is visualized in Supplementary Figure S3. The output of each Proteinarium run was analyzed for the presence of significant clusters (*p* < 0.05) dominated by MMR-intact patients. The MMR-intact patients in the cluster with the largest number of MMR-intact patients and less than two MMR-deficient patients were compiled in a list of “selected” MMR-intact patients. These MMR-intact-dominated clusters contained patients with significant PPI network similarity. After running 15 iterations of the randomly chosen MMR-intact versus MMR-deficient patients in Proteinarium, the list of selected MMR-intact patients contained 32 patients. Finally, Proteinarium was used to compare the 32 selected MMR-intact patients with the 29 MMR-deficient patients. The output of Proteinarium yielded a dendrogram and list of significant clusters that were used in subsequent analyses.

### 4.3. Separation Test

Separation testing was used to determine the genetic similarity between diseases or phenotypes of diseases by comparing their PPI networks in the interactome. We performed a separation test to determine the distance between the PPI networks of the MMR-deficient- and MMR-intact-dominated clusters using a Python script adapted from Menche et al. [57]. The interactome is composed of 141,296 experimentally determined physical interactions between 13,460 proteins. S_AB_ is a measure of the distance between two diseases in the interactome, with a positive value indicating a topological separation of the disease modules and a negative value indicating that the diseases overlap in the interactome.

### 4.4. Statistical Analysis

Comparative analysis and Kaplan–Meier survival analysis were performed using variable statistical methods provided in the cBioPortal user interface. Kaplan–Meier survival plots were made to demonstrate survival curves in relation to time in months by the log-rank methods.

## 5. Conclusions

In summary, the present study demonstrates MMR-deficient BC to be a distinct molecular subtype with unique PPI networks involving histone hub genes and variable clinical significance depending on a patient’s individual HR status. Molecular subtyping based on MMR could be important for characterizing tumors as MMR-deficient and guiding the use of immune checkpoint inhibition and other targeted therapies in both TN as well as HER2-enriched tumors.

## Data Availability

All data collected and analyzed in this study are available at cBioPortal (https://bit.ly/3g4lTM4) (accessed on 1 February 2022).

## Supplementary Materials

The following supporting information can be downloaded at: https://www.mdpi.com/article/10.3390/ijms24065327/s1.
