# Supplementary material for: MMR Deficiency Defines Distinct Molecular Subtype of Breast Cancer with Histone Proteomic Networks
Source: Int J Mol Sci. 2023 Mar 10;24(6):5327. doi: 10.3390/ijms24065327 (PMC10049366; doi:10.3390/ijms24065327)
Supplement: Supplementary file 1 [file ijms-24-05327-s001.zip › Supplementary_Figure_Legends.pdf]

Supplementary Figure S1. Survival analyses across different molecular breast cancer subtypes for MMR mutated tumors. Luminal B (Excluding VUS): (a) progression-free survival; (b) overall survival; (c) disease-specific survival. HER2 (Excluding VUS) (d) progression-free survival; (e) overall survival; (f) disease-specific survival. HER2 (Including VUS) (g) disease-free survival; (h) progression-free survival; (i) disease-specific survival. Basal/TN (Including VUS) (j) overall survival; (k) disease-specific survival.
The number of patients at risk in each group: Disease-free survival, 2 MMR deficient patients at risk of event; Progression-free survival, 4 MMR deficient patients at risk of event; Overall survival, 4 MMR deficient patients at risk of event; Disease-specific survival, 3 MMR deficient patients at risk of event.

Supplementary Figure S2. Cophenetic correlation coefficients. Cophenetic correlation coefficients comparing the dendrograms produced by comparing the dendrogram output of Proteinarium comparisons using the top 60, 100, 150, 200, 250, and 300 genes as seed gene inputs.

Supplementary Figure S3: Random iteration workflow. The output of each Proteinarium run was analyzed for the presence of significant clusters (p-value < 0.05) dominated by MMR intact patients. The MMR intact patients in the cluster with the largest number of MMR intact patients and less than two MMR deficient patients was compiled in a list of "selected" MMR intact patients. These MMR intact-dominated clusters contained patients with significant PPI network similarity. After running 15 iterations of the randomly chosen MMR intact versus MMR deficient patients in Proteinarium, the list of selected MMR intact patients contained 32 patients. Finally, Proteinarium was used to compare the 32 selected MMR intact patients with the 29 MMR deficient patients. The output of Proteinarium yielded a dendrogram and list of significant clusters that were used in subsequent analyses.
