# Supplementary material for: MMR Deficiency Defines Distinct Molecular Subtype of Breast Cancer with Histone Proteomic Networks
Source: Int J Mol Sci. 2023 Mar 10;24(6):5327. doi: 10.3390/ijms24065327 (PMC10049366; doi:10.3390/ijms24065327)
Supplement: Supplementary file 1 [file ijms-24-05327-s001.zip › supplementary_tables_corrected.pdf]

Supplementary Tables

Supplementary Table S1. Number of MMR deficient patients in each molecular subtype.

MMR gene	Patients (%)	Truncating	Splice	SV/fusion	Missense	CNA	VUS
MSH2	4 (14%)	1	1	2			
MSH3	10 (34%)	3				7	5 missense
MSH6	3 (10%)	2	1				8 missense
MLH1	8 (28%)	2	1	1	1	4	5 missense
PMS1	4 (14%)	2		1		1	9 missense
MLH3	0 (0%)						11 missense, 1 in-frame
PMS2	3 (10%)	2				1	6 missense

* SV, structural variants; CNA, copy-number alterations.

Supplementary Table S2. Overview of MMR mutations.

MMR Gene	Cohort Including VUS (n=89)	Cohort Excluding VUS (n=29)
PMS1	18	4
PMS2	14	2
MLH3	10	0
MSH2	10	4
MSH3	9	7
MLH1	8	7
MSH6	5	2
MSH2, MSH6	5	0
MLH1, MSH2, MSH3, MSH6	1	0
MLH1, MLH3, MSH6	1	0
MSH3, PMS1	1	0
MLH1, MSH3	1	1
MLH3, MSH6	1	0
MLH1, PMS1	1	0
MLH1, MLH3	1	0
MLH3, PMS1	1	0
MSH3, PMS2	1	1
MSH3, MSH6	1	1

Supplementary Table S3. Comparisons between MMR deficient and intact patients excluding VUS.

Clinical Attribute	Statistical Test	p-value	q-value
Mutation Count	Wilcoxon Test	**1.29e-7**	**2.121e-6**
TMB (nonsynonymous)	Wilcoxon Test	**3.27e-7**	**3.928e-6**
Tumor Type	Chi-squared Test	**2.289e-6**	**2.197e-5**
MSIsensor Score	Wilcoxon Test	**8.180e-6**	**4.908e-5**
MSI MANTIS Score	Wilcoxon Test	**1.275e-4**	**6.472e-4**
Molecular Subtype	Chi-squared Test	**2.061e-4**	**8.242e-4**
Diagnosis Age	Wilcoxon Test	0.212	0.528
Radiation Therapy	Chi-squared Test	0.357	0.815
Fraction Genome Altered	Wilcoxon Test	0.427	0.851
Neoadjuvant Therapy Type	Chi-squared Test	0.429	0.851
Aneuploidy Score	Wilcoxon Test	0.486	0.864
Ethnicity Category	Chi-squared Test	0.659	0.999
Sex	Chi-squared Test	0.746	0.999
Metastasis	Chi-squared Test	0.806	0.999
Race Category	Chi-squared Test	0.932	0.999
Tumor Stage	Chi-squared Test	0.962	0.999
Prior Diagnosis	Chi-squared Test	0.971	0.999
Lymph Node Stage	Chi-squared Test	0.983	0.999
AJCC Stage	Chi-squared Test	0.993	0.999

*TMB, Tumor Mutational Burden; MSI, Microsatellite instability; AJCC, American Joint Committee on Cancer.
