# Supplementary figures and images for: MMR Deficiency Defines Distinct Molecular Subtype of Breast Cancer with Histone Proteomic Networks
Source: Int J Mol Sci. 2023 Mar 10;24(6):5327. doi: 10.3390/ijms24065327 (PMC10049366; doi:10.3390/ijms24065327)

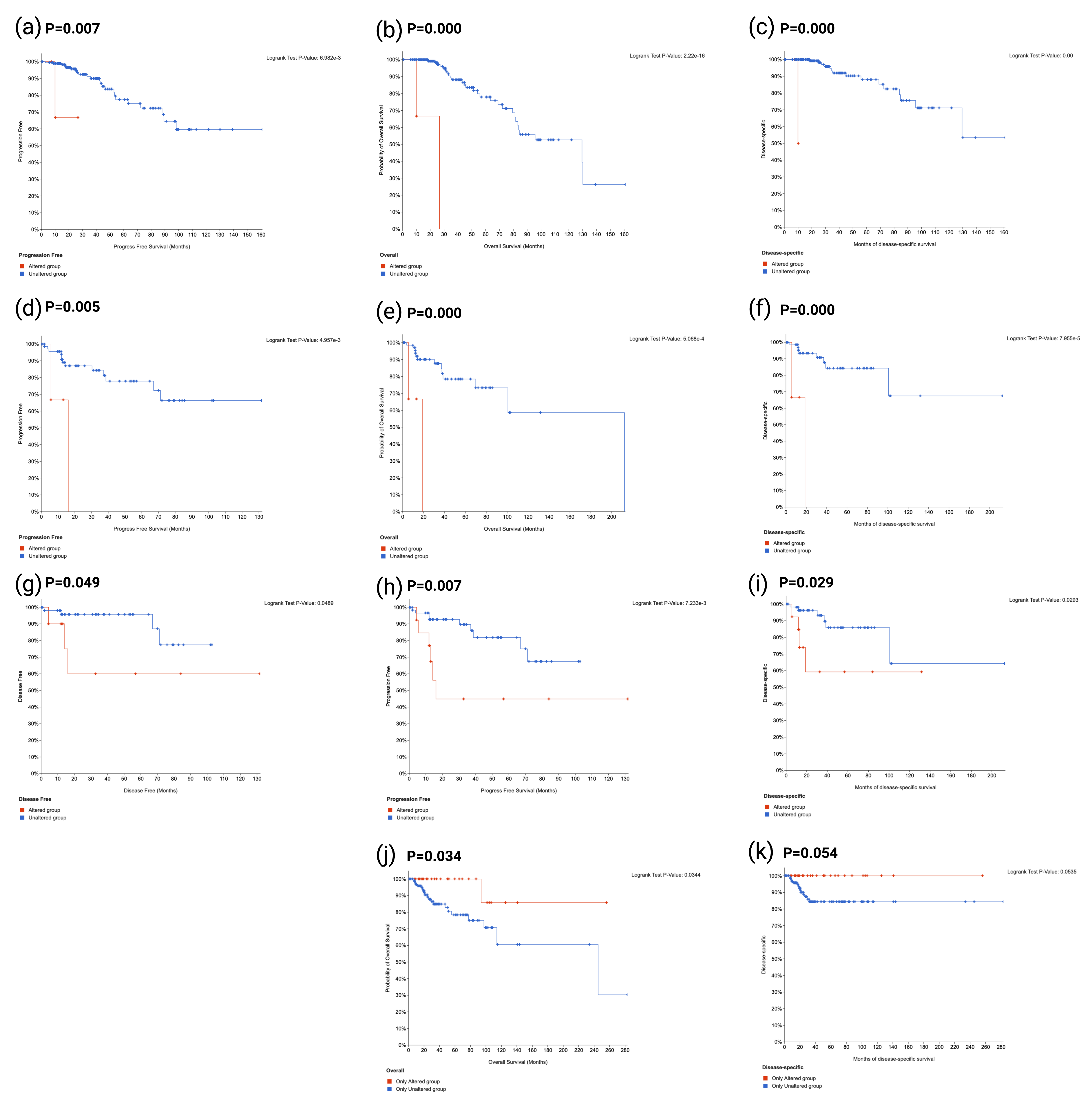

Supplement: Supplementary file 1 [file ijms-24-05327-s001.zip › Supplementary_Figure1_updated.png]

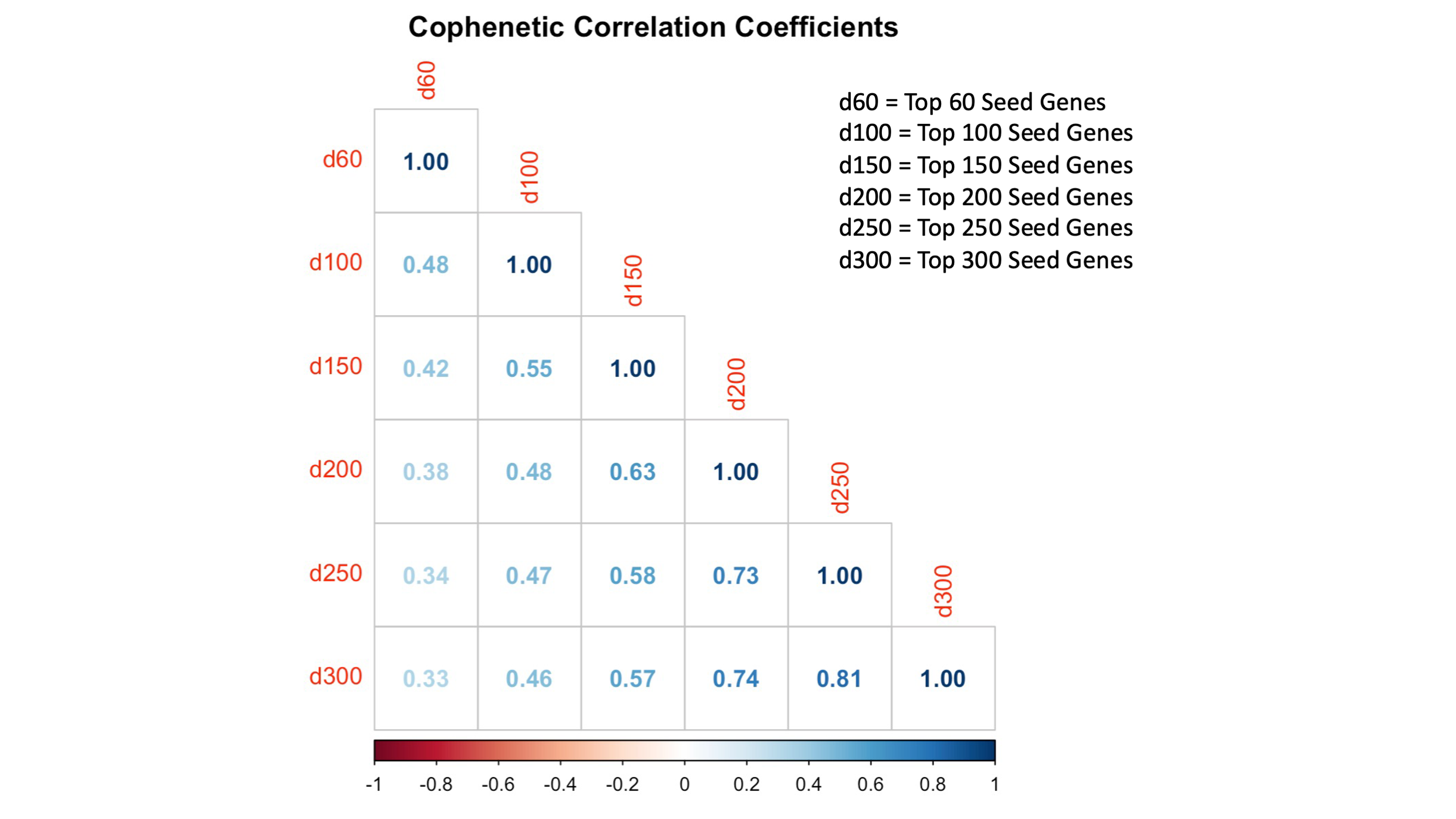

Supplement: Supplementary file 1 [file ijms-24-05327-s001.zip › Supplementary_Figure2_updated.tiff]

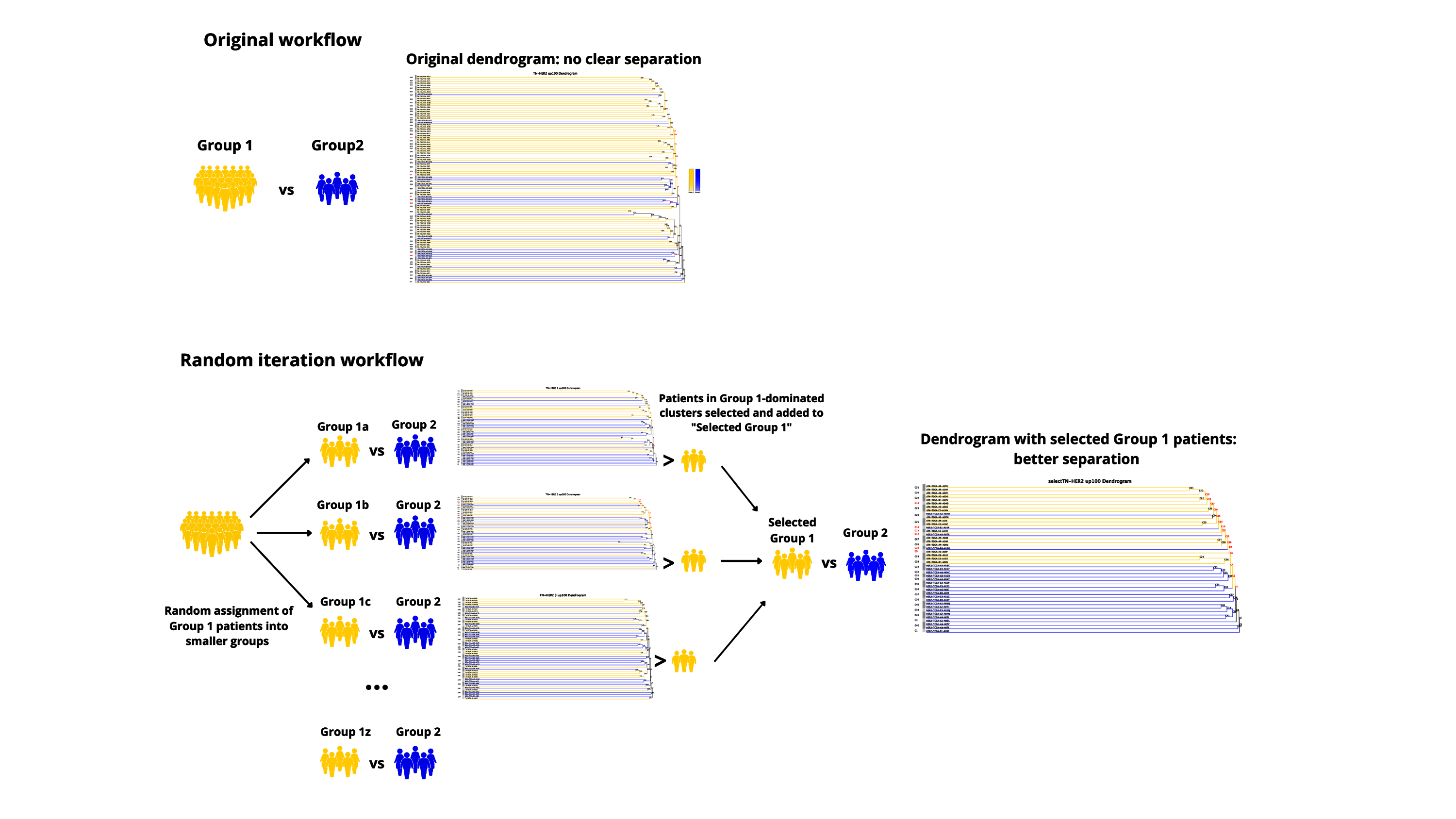

Supplement: Supplementary file 1 [file ijms-24-05327-s001.zip › Suppmentary_Figure3_updated.tiff]
